# Carcinoid Heart Disease Revealing the Burden of a Neuroendocrine Tumour: A Case Report

**DOI:** 10.7759/cureus.93721

**Published:** 2025-10-02

**Authors:** Rahma Nazar, Diya Devassy, Dabeet Sajeev, Sujita Baidya

**Affiliations:** 1 General Medicine, Maidstone and Tunbridge Wells NHS Trust, Kent, GBR; 2 General Internal Medicine, Royal Derby Hospital, Derby, GBR

**Keywords:** carcinoid heart disease, carcinoid syndrome, echocardiogram (echo), heart failure, neuroendocrine tumour, tricuspid regurgitation

## Abstract

Carcinoid heart disease (CHD) is a rare but serious complication of carcinoid syndrome (CS), typically arising in patients with metastatic neuroendocrine tumours (NETs). Prolonged exposure of the right side of the heart to vasoactive substances such as serotonin leads to progressive valvular fibrosis, predominantly affecting the tricuspid and pulmonary valves, and often culminates in right-sided heart failure. We report the case of a 67-year-old woman with a metastatic small bowel NET who developed CS and later presented with worsening exertional dyspnoea and peripheral oedema. Echocardiography demonstrated severe tricuspid regurgitation and moderate pulmonary regurgitation with preserved left ventricular systolic function. Despite aggressive diuretic therapy, her condition deteriorated, and she developed refractory right-sided heart failure with generalised anasarca. She was not a candidate for valve replacement or further disease-directed therapy due to advanced metastatic disease and frailty and was therefore managed palliatively until she passed away. This case highlights the severe burden of CHD in patients with serotonin-secreting NETs and emphasises the importance of early recognition and regular echocardiographic surveillance to facilitate timely intervention and potentially improve outcomes.

## Introduction

Carcinoid tumours are slow-growing neuroendocrine neoplasms arising from enterochromaffin (EC) cells, most commonly located in the gastrointestinal tract, particularly the appendix and terminal ileum. Less frequently, they may arise in the bronchus or gonads. The reported incidence is 2.5 to 5.0 cases per 100,000 individuals annually [1]. A well-recognised complication is carcinoid syndrome (CS), which develops in about 30-40% of patients with metastatic disease [2]. Once CS develops, approximately half of these patients progress to carcinoid heart disease (CHD), particularly in the presence of hepatic metastases that permit vasoactive substances to bypass first-pass metabolism in the liver [3].

The cardiac manifestations of CHD are not caused by direct tumour metastasis but by the paraneoplastic effects of vasoactive substances such as serotonin, histamine, tachykinins, and prostaglandins released by malignant cells. Normally, these products are inactivated by the liver. However, hepatic metastases allow large quantities to reach the right side of the heart, leading to fibrotic thickening and dysfunction of the endocardium and cardiac valves, especially the tricuspid and pulmonary valves [4]. CHD is associated with poor prognosis, with mean survival reduced to 1.6 years compared to 4.6 years in those without cardiac involvement [5]. Elevated urinary 5-hydroxyindoleacetic acid (5-HIAA), the breakdown product of serotonin, provides a key diagnostic marker of CS and CHD. Here, we present the case of a patient with a neuroendocrine tumour (NET) complicated by CHD, progressing to severe valvular dysfunction, highlighting the importance of early diagnosis through echocardiographic surveillance, which may help prevent such advanced cardiac involvement.

## Case presentation

A 67-year-old woman with a history of hypertension and chronic kidney disease was diagnosed with a metastatic NET of the small bowel five years ago, consistent with stage IV disease. At the time of diagnosis, her baseline urinary 5-HIAA level was 200 µmol/24 hours. She developed CS two years later and had been receiving subcutaneous Lanreotide injections every 28 days for the past three years. She presented with progressively worsening exertional shortness of breath over six months and bilateral leg swelling. District nurses, who had been reviewing her at home, noted new bibasal crepitations on auscultation, which prompted referral to the hospital for further evaluation.

**Figure 1:**
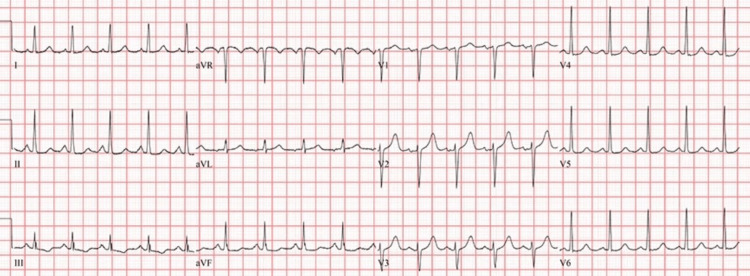
Electrocardiogram Electrocardiogram shows sinus tachycardia.

**Figure 2:**
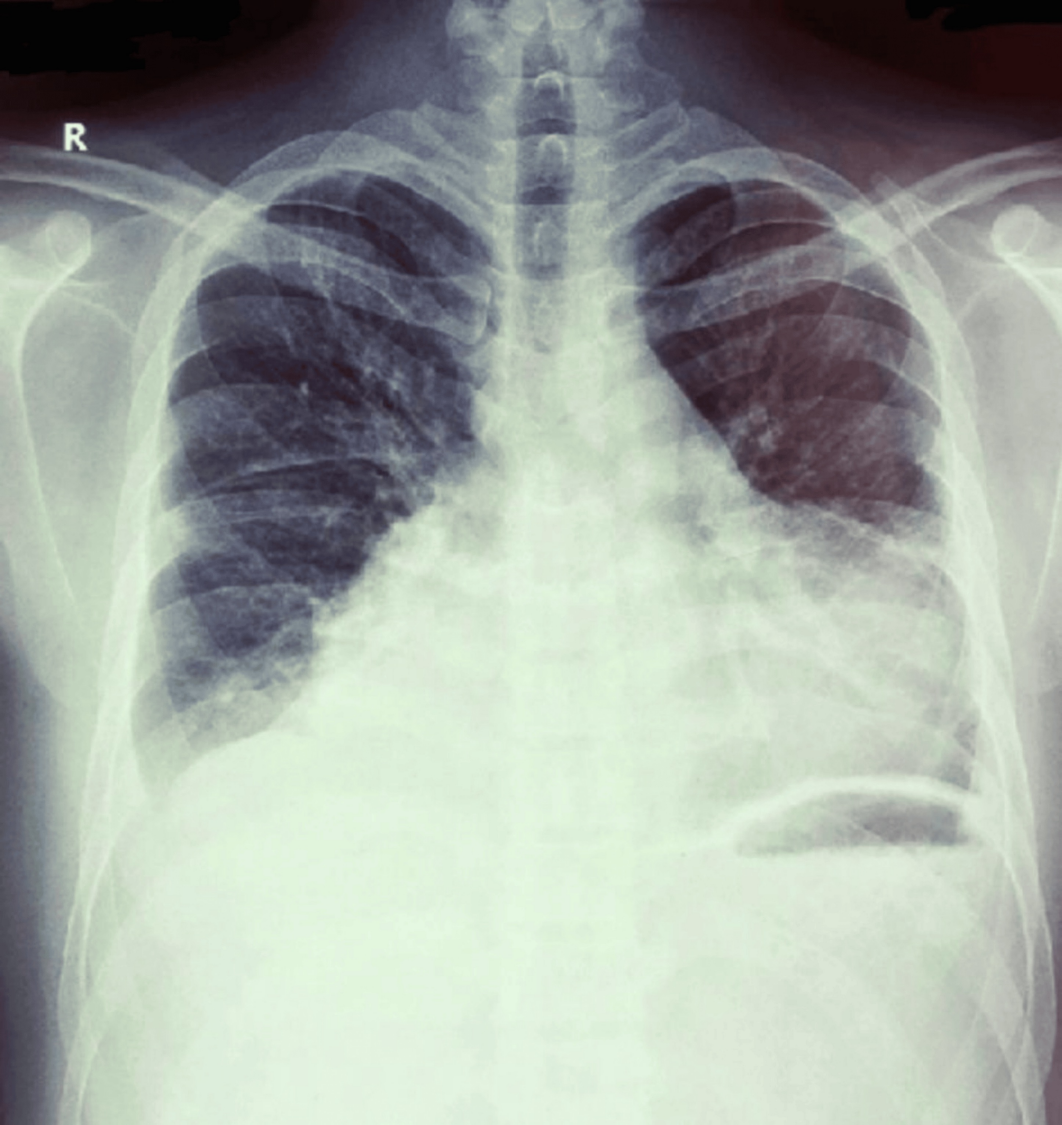
Chest X-ray Chest X-ray shows cardiomegaly with bilateral pleural effusions.

On arrival, her vital signs were oxygen saturation 85% on room air, heart rate 120 beats per minute, blood pressure 130/80 mmHg, respiratory rate 30 breaths per minute, and temperature 37°C. Examination findings included a pansystolic murmur most prominent at the left middle sternal border, bibasal lung crepitations, bilateral pitting oedema extending up to the abdomen, and raised jugular venous pressure. Laboratory investigations revealed normal inflammatory markers and hypokalaemia, likely secondary to poor nutritional intake from her symptoms (Table 1). ECG demonstrated sinus tachycardia (Figure 1), and chest radiography revealed cardiomegaly with bilateral pleural effusions (Figure 2).

**Table 1:** Initial Laboratory Panel mg/L: milligrams per litre, mmol/L: millimoles per litre, umol/L: micromoles per litre, g/L: grams per litre.

Parameters	Findings	Normal Range
C-Reactive Protein	3mg/L	<5mg/L
Sodium	140mmol/L	133-146mmol/L
Potassium	2.7mmol/L	3.5-5.3mmol/L
Creatinine	80umol/L	44-80umol/L
Haemoglobin	110g/L	120-150g/L
White Cell Count	6.34x10⁹/L	4.00-10.00x10⁹/L
Platelet Count	387x10⁹/L	150-410x10⁹/L

**Figure 3:**
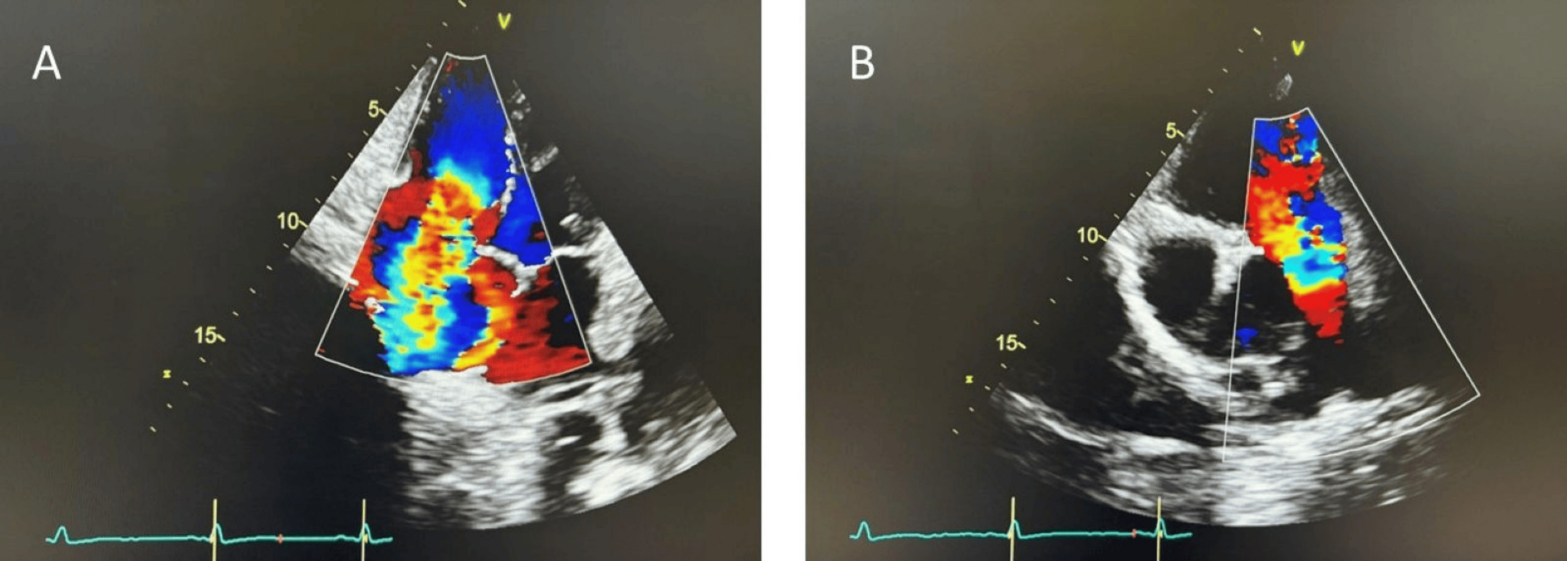
Transthoracic Echocardiography A) Apical four-chamber view showing severe free flow tricuspid regurgitation. B) Parasternal short-axis view showing moderate pulmonary regurgitation.

She was initially managed with oxygen therapy and intravenous furosemide 40 mg twice daily alongside potassium replacement. Transthoracic echocardiography (TTE) demonstrated severe tricuspid regurgitation, moderate pulmonary regurgitation, and low-normal left ventricular systolic function (visual ejection fraction 50-55%). The right heart was enlarged, with a dilated right ventricle (RVD1 49 mm, RVD2 47 mm) and a dilated right atrium (RA area 28 cm²) (Figure 3).

Due to persistent symptoms, the cardiology team escalated management to a continuous furosemide infusion (160 mg/24 hours) and added spironolactone, with careful monitoring of fluid balance, electrolytes, and blood pressure. She initially responded with improved oxygenation and reduced oedema; however, she developed symptomatic postural hypotension, necessitating temporary cessation of diuretics.

During her hospital stay, her condition continued to decline, with the development of ascites and generalised anasarca despite optimisation of medical therapy. The NET team deemed her unsuitable for further disease-directed therapy, and she was not considered a candidate for valve replacement in view of advanced metastatic disease and frailty. Palliative care input was sought, and she was managed with anticipatory medications and palliative oxygen for symptomatic relief.

She remained an inpatient throughout her admission and, after 35 days in hospital, passed away with right-sided heart failure secondary to CHD as the primary contributor.

## Discussion

CHD, also known as Hedinger syndrome, is a unique and unfortunate complication of CS [6]. CS arises most commonly in midgut NETs involving the jejunum, ileum, cecum, and ascending colon, while tumours in the foregut or hindgut are less likely to cause the syndrome. This association is explained by the high density of EC cells in the midgut. These specialised neuroendocrine cells produce and release serotonin, a key regulator of intestinal motility and secretion, which explains why carcinoid syndrome, and subsequently CHD, is strongly linked to midgut NETs rather than NETs at other sites.

The interplay of bioactive substances such as serotonin and other vasoactive agents leads to the characteristic fibrotic changes predominantly in the right-sided heart valves [7]. The valvular fibrosis observed in CHD is a direct consequence of persistent serotonin exposure, which is normally inactivated in the lungs and liver. In the presence of hepatic metastases, this detoxification pathway is bypassed, allowing large amounts of serotonin to reach the right-sided heart structures [8]. This results in the formation of endocardial fibrous plaques, not only in the valve leaflets but also in the subvalvular apparatus, including tendinous chords and papillary muscles. Although valve morphology may remain intact, endocardial thickening causes retraction and fixation, leading to severe regurgitation. While serotonin is considered a major initiator of the fibrotic process, the pathophysiological mechanisms remain incompletely understood, and other tumour-secreted factors are also believed to contribute [9].

Metastatic carcinoid tumours take up tryptophan and convert it to serotonin, which is ultimately metabolised to 5-HIAA, accounting for over 95% of urinary serotonin breakdown products (Figure 2). Patients with carcinoid syndrome therefore demonstrate elevated 24-hour urinary 5-HIAA [4]. Some studies have demonstrated a positive correlation between urinary 5-HIAA levels, disease progression, and worsening prognosis. This has been attributed to the fact that higher circulating concentrations of vasoactive substances (particularly serotonin, which induces fibroblast proliferation) are more likely to result in progressive cardiac damage [10,11]. 

**Figure 4:**
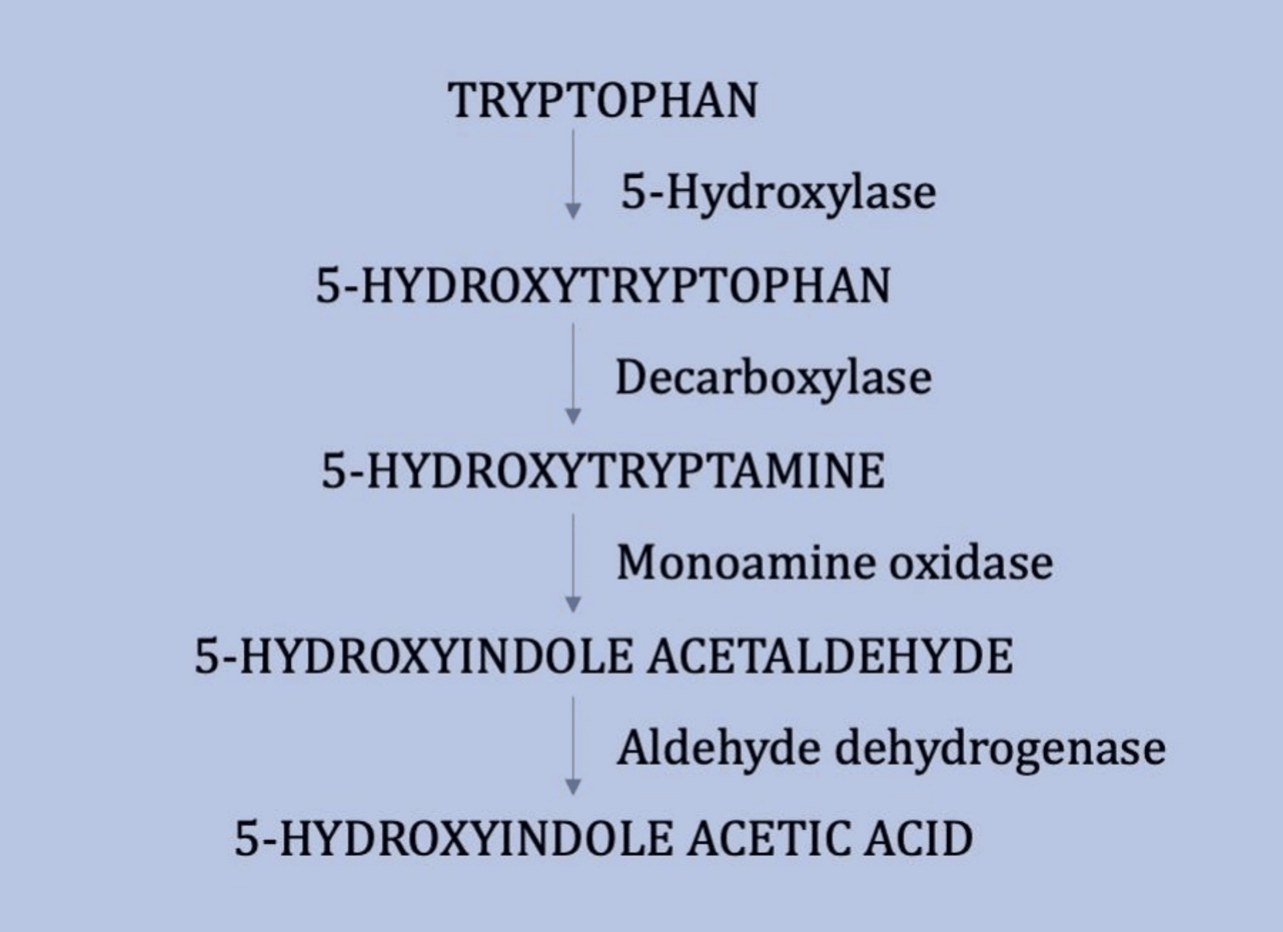
Biochemical Pathway for the Synthesis and Degradation of Serotonin (5-Hydroxytryptamine) Information adapted from reference [4]. Used with permission from the BMJ Publishing Group Ltd. License number: 6100130409812.

The clinical manifestations of CHD reflect valvular heart disease and right-sided heart failure. These include dyspnoea, ascites, peripheral oedema, pleural effusions, pulsatile hepatomegaly, and elevated jugular venous pressure with prominent V waves. A pansystolic murmur at the left middle sternal border, corresponding to tricuspid regurgitation, is common, while murmurs of pulmonary stenosis or regurgitation may also be detected. However, these findings may be subtle, and symptoms often nonspecific, making a high index of suspicion crucial for timely diagnosis.

TTE remains the gold standard for diagnosis and should ideally be performed at the time of diagnosing serotonin-producing NETs and repeated annually [11]. Management of CHD involves a multifaceted approach, including treatment of right heart failure with cautious diuresis and salt restriction, pharmacological therapy to reduce tumour secretion of vasoactive substances (e.g., somatostatin analogues), which has been associated with improved symptoms and survival, and surgical/interventional valve replacement, which remains the gold standard for addressing advanced valvular pathology, more recently, transcatheter valve interventions have emerged as a potential option in selected inoperable or frail patients [12].

A recent study by Fijalkowski et al. highlighted that CHD may not present with symptoms initially; however, echocardiographic valve alterations such as tricuspid insufficiency can be detected early, enabling surgical intervention and potentially prolonging survival [13,14]. In our case, the patient’s CHD was only recognized once severe right-sided heart failure had developed, by which point she was not a candidate for surgical management. This case therefore suggests that early diagnosis with echocardiographic surveillance of CHD in patients with NETs may be beneficial, allowing timely interventions before irreversible cardiac damage occurs.

## Conclusions

This case demonstrates the severe burden that can arise in patients with NETs when complicated by CHD. Despite advances in tumour-directed therapy and availability of somatostatin analogues, cardiac involvement continues to be a major determinant of morbidity and mortality. The progression to advanced right-sided heart failure in this case highlights the importance of early recognition and regular echocardiographic surveillance in patients with serotonin-producing tumours. Early detection of valvular pathology may allow timely intervention and improve clinical outcomes.
